# Spatial analysis of healthcare services availability and demand for people aged 65 and over in Québec

**DOI:** 10.1007/s43999-026-00085-5

**Published:** 2026-01-21

**Authors:** Juliette Duc, Nevena Veljanovic, Sébastien Barbat-Artigas, David L. Buckeridge, Delphine Bosson-Rieutort

**Affiliations:** 1 Department of Health Management, Evaluation and Policy, School of Public Health, Université de Montréal, Montréal, Québec Canada; 2 Centre de recherche en santé publique (CReSP), Université de Montréal et Centre intégré universitaire de santé et de services sociaux du Centre-Sud-de-l’Île-de-Montréal, Montréal, Québec Canada; 3 Centre Interuniversitaire de Recherche en Analyse Des Organisations (CIRANO), Montréal, Québec Canada; 4 Unité d’évaluation des technologies et modes d’intervention en santé et services sociaux (UETMIS-SS), Direction des affaires universitaires, de l’enseignement et de la recherche (DAUER), Centre intégré universitaire de santé et de services sociaux de l’Ouest-de-l’île-de-Montréal (CIUSSS-ODIM), Montréal, Québec Canada; 5 Department of Epidemiology, Biostatistics and Occupational Health, McGill University, Montréal, Québec Canada

**Keywords:** Healthcare disparities, Health services needs and demand, Aged population, Urbanization, Québec, Spatial analysis

## Abstract

**Background:**

As people age, their healthcare needs increase and become more complex, requiring a corresponding increase in healthcare and services use. Moreover, heterogeneity of healthcare needs and availability can be observed among the health regions within Canadian provinces, especially between rural and urban regions. The province of Québec has received limited attention in this regard. This study aims to describe and compare healthcare services location and aging population healthcare demand across Québec.

**Methods:**

We used data from *Données Québec* to describe the distribution of available healthcare (such as facilities, their services and capacity) and potential demand of services (represented by the location of the aged population) and mapped their relationship based on urbanization level. Analyses were performed using QGIS and R software.

**Results:**

We found a substantial variability of the population aged 65 and over, the number of facilities, the number and type of services, and long-term care (LTC) beds between regions in Québec. The number of LTC beds was significantly correlated with the number of people aged 65 and over (R² = 0.88, *p* < 0.001), but not with their proportion. LTC accommodation is a service most offered in urban areas, especially in the Montréal region.

**Conclusion:**

Healthcare availability in Québec is associated with the number of older people and there tend to be fewer services, including LTC, overall and per older person in rural as compared to urban areas. Variability of the number of facilities and services provided was higher at the local scale.

**Supplementary Information:**

The online version contains supplementary material available at 10.1007/s43999-026-00085-5.

## Introduction

As observed in all OECD countries, a demographic shift is occurring in Canada, so that by 2031 over 25% of the population will be aged 65 years and over in Québec [1]. The aging of the population raises important healthcare concerns as most individuals experience an accumulation of health problems and more specific and complex needs as they age [2]. In Canada, the prevalence of multiple chronic diseases has been increasing from 30% for the 40–49 years old population to 52% for the 60–64 years old population [3]. Specifically, in Québec in 2016–2017, 45% of individuals aged 65 and over were living with at least two diagnosed chronic diseases [4]. Thereby, aging individuals may face multiple health problems, leading to increased use of healthcare services until their death [5], as demonstrated in Ontario where total service use was 2.5 times higher for those aged 85 and over than for those aged 65–69 [6].

The increasing prevalence of multiple chronic illnesses implies that people’s needs are becoming more complex. This complexity of needs can reflect not only health-related characteristics or utilization, but also cultural, socioeconomic and environmental factors [7]. For example, complexity may reflect seeking care from multiple providers. This complexity results in significant heterogeneity in the health status of the population and the use of healthcare services [5, 8] with variation observed across regions [9]. For instance, there is an approximately 4% difference in the prevalence of multimorbidity between certain regions of Québec [4]. In parallel, frailty has emerged as a distinct but related concept capturing age-related biological declines associated with negative health-related outcomes [10], and has been shown to be associated with increased healthcare service use and higher mortality rates [11]. The increasing prevalence of frailty in aging populations further complicates care needs and underscores the importance of considering both medical and functional vulnerability in healthcare planning. Therefore, the combination of medical complexity and increasing frailty implies that older adults may have a wide range of needs. It is crucial to adapt the healthcare system to meet the needs of the aging population through better resource management and timely delivery of appropriate services [12]. However, the required adaptation of healthcare systems has been a major challenge for several years [13]. Inappropriate use of services and difficulties in meeting patient demands are common issues that can impact the quality of life of older individuals and increase healthcare system costs [14, 15]. It is consequently essential to consider all the elements surrounding patients to better understand the overall healthcare use by the aging population.

A key element to consider when studying healthcare utilization is the availability of services. According to Penchansky and Thomas [16], it is one of the five dimensions that can affect the transition from potential access to care to its realization and corresponds to the number and type of local service points available to a patient in their environment. Heterogeneity in healthcare availability can be observed in several large territories worldwide, which can result in inadequate management of patients with complex needs in their region of residence, leading them to seek care in other regions. A study conducted in Manitoba demonstrated that a higher ratio of beds and doctors per patient was associated with a reduced likelihood of hospitalization outside the region. However, 20% of hospitalizations of people aged 65 and over in the six months before death occurred outside their region of residence [17]. Hospitalization outside one’s regions of residence can have serious consequences for older adults as they may receive less family support, be subject to more expenses, and face problems in communicating and transmitting medical information [18, 19]. These findings highlight the potential lack of resources required to meet patients’ needs within the regions of residence [20] and therefore the possibility that variations in healthcare availability may not align with variations in demand. For instance, a study in Saskatchewan demonstrated a discrepancy between the number and distribution of physiotherapists and the healthcare needs of the population [21]. However, there has been no study to date of the overall services in the province of Québec. This gap is particularly notable given that Québec is distinct among Canadian provinces in its integration of healthcare and social services within a single public system framework.

These variations in the healthcare availability between regions depend on the characteristics of those regions, particularly their urbanization level. Indeed, urbanization can affect both the healthcare availability and its utilization, and is important to consider in Canada as nearly 20% of the population resides in rural areas, a proportion reaching 25% in Québec [22]. In Manitoba, rural residents reportedly have less access to appropriate health resources for end of life care than urban residents, with fewer doctors available in rural areas [9]. A study in the United States also showed that living in rural areas negatively affected access to the healthcare system due to a lower number of Medicare-certified hospices in these areas [23]. Differences in health resource availability between rural and urban settings may contribute to variation in healthcare utilization. For example, decreased physician visits by residents of small urban or rural areas compared to large urban areas, despite similar health needs, have been demonstrated in Saskatchewan [24]. It is also important to note that, in Canada, rural areas tend to have a higher proportion of residents aged 65 and over than urban areas and are, proportionately, aging faster [25, 26]. This demographic profile adds further pressure on local healthcare systems.

In summary, there appears to be a discrepancy between healthcare availability and population’s needs between regions, especially when considering urbanization level. However, as previously stated, while several studies have focused on specific Canadian provinces, Québec has received limited attention, partially because of language and data-access barriers [27, 28]. Specifically, there is a lack of investigation into whether the distribution of available healthcare adequately matches the aging population health needs, especially in rural areas. This work aims to describe and compare the variations of healthcare services and aging population health demand in Québec. Our objectives were to (1) map the relationship between the location of healthcare services and the population aged 65 and over in Québec, and (2) study these distributions according to the urbanization level. We anticipated that highly urban areas will have more healthcare facilities and services, while rural areas may have a difference between healthcare availability and demand in older populations.

## Methods

### Data sources

#### Geographic boundary

The province of Québec is divided into 18 health regions (*régions sociosanitaires*, RSS) encompassing 93 local health networks (*réseaux locaux de services*, RLS), subsequently also referred to as regional or local scale respectively. We used the 2024 cartographic boundary files available on the “Données Québec” website (www.donneesquebec.ca). As data were missing for the regions of Nunavik, Terres-Cries-de-la-Baie-James and Nord-du-Québec, these regions were excluded from the analyses.

#### Characteristics and location of available healthcare

In Québec, healthcare establishments are the legal entities responsible for managing healthcare and social services [29]. These establishments operate one or several facilities, which are the physical places where services are provided to the Québec population. Each facility is assigned one or more missions, based on the services they provide, that can be categorized into 5 groups: long-term care centres (LTC), local community services centres (CLSC), hospital centres (HC), rehabilitation centres (RC) and child and youth protection centres (CPEJ). A facility may offer multiple services, such as facilities whose mission is “hospital centres” that can provide both “general and specialized care” and “psychiatric care” services. Since this study concerned people aged 65 and over, we focused on, hospital centres, emergency rooms, family medicine groups, daycare centre, CSLCs and LTC services, referring to residential long-term care facilities that provide 24-hour nursing care, personal assistance, and psychosocial support to individuals with significant loss of autonomy, primarily older adults. Other services were categorized as “Other”. Data were obtained from the cartographic boundary files of healthcare establishments and facilities of April 2024, provided and updated monthly by the Ministry of Health and Social Services (“Ministère de la Santé et des Services sociaux”, MSSS) on the “Données Québec” website (www.donneesquebec.ca). Data used included geographical location of establishments and facilities, service(s) provided, and capacity when relevant (number of beds or places within each service). We aggregated results to the local health networks level to facilitate interpretation of maps.

#### Population data

We used the number of people aged 65 and over as a proxy of healthcare potential demand, assuming their direct proportionality. This assumption is supported by evidence that age is associated with increased healthcare needs and that older adults account for a disproportionately large share of health-care use in Canada [3, 30]. These measures were calculated using a 2024 projection based on the Québec 2022 data census provided by Statistics Canada and the Québec institute of statistic (“Institut de la Statistique du Québec”) on the “Données Québec” website (www.donneesquebec.ca). These data are available by sex and age group and were disseminated according to the hierarchical territorial divisions of the health network. As this study focused on older adults, we selected data related to people aged 65 and over who were living in Québec.

#### Rural and urban areas

The term urban usually refers to a region characterized by a high population concentration (> 1,000 inhabitants) and a high density (> 400 inhabitants per square kilometer). An urban region is defined as a small, medium or large population centre depending on its population (1,000–29,999; 30,000–99,999 and 100,000+). Regions that do not meet these criteria are called rural [31]. The 2021 urbanization index was provided by Statistics Canada at the dissemination area and blocks scale. The dissemination area is the smallest geographic scale that contains at least 1,000 individuals. The designation “rural” was assigned to each local health networks if at least 50% of its dissemination areas were “rural” according to Statistics Canada. In addition, an additional categorical variable as “urban”, “mid urban”, “mid rural” and “rural” has been created using the 25%, 50% and 75% cut-off.

### Data management, spatial and statistical analysis

We computed metrics on healthcare availability, population distribution, and urbanization at both the regional and local levels. For the spatial representation, georeferenced shapefiles (geographic boundaries and facility locations) were imported and overlaid. Other datasets (population, healthcare offer characteristics and urbanization levels) were integrated through attribute-based joins using territory identifiers. We chose to present the results at the local health networks scale which offers the best compromise between details and information completeness. Colour gradations were used to display either the population or the urbanization level, whereas dots and pie charts were used to indicate the location of health facilities, establishments, services, and capacity. Parametric statistical tests were used for variables with normal distribution (Student t-test and ANOVA), otherwise non-parametric statistical tests were used (Wilcoxon test and Kruskal-Wallis test). All data management, statistical analysis and plots production were performed using R software [32], and maps were generated using QGIS software [33].

## Results

### Demand and healthcare location

**Fig. 1:**
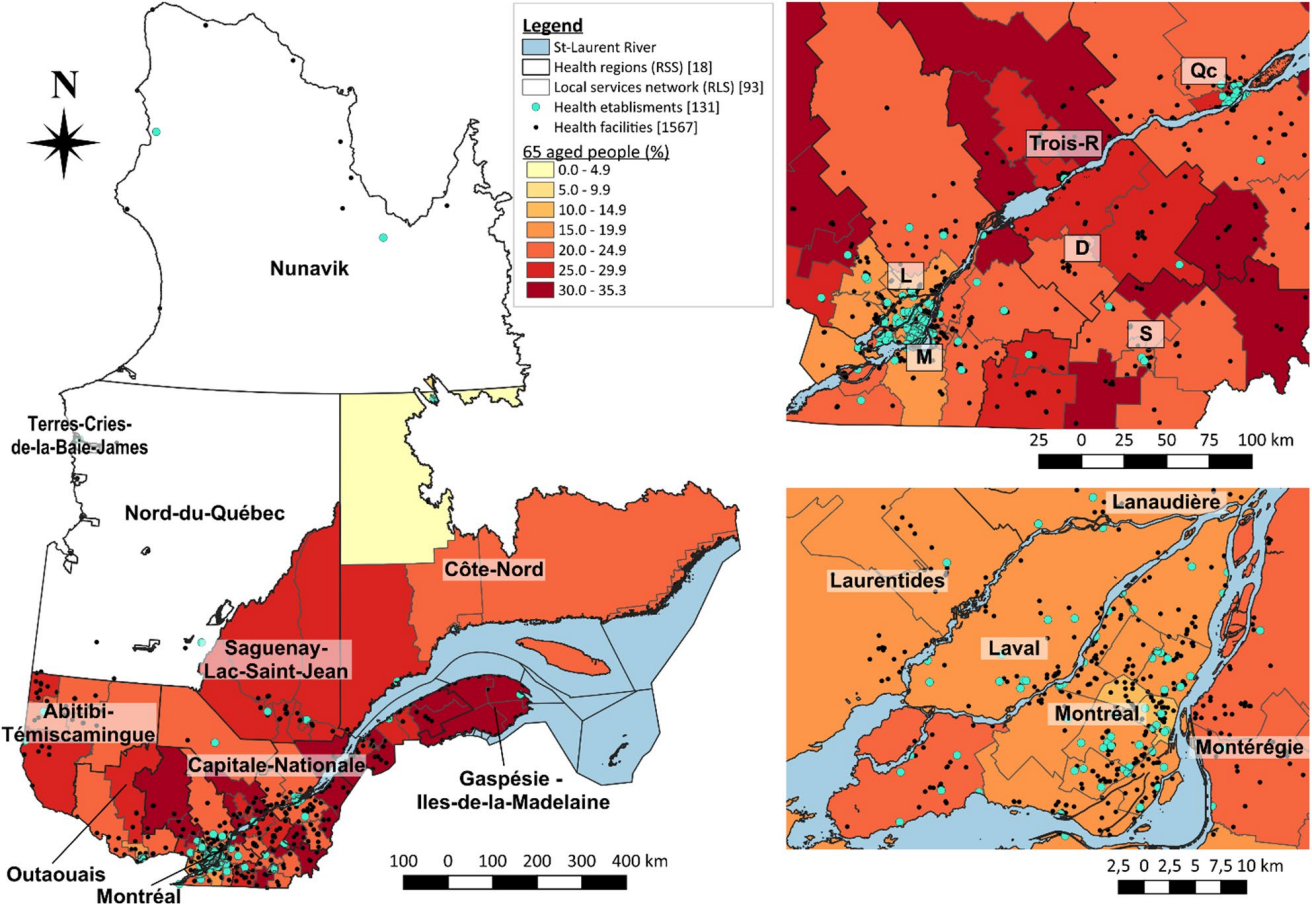
Proportion of people aged 65 and over and facilities location at local health network scale. *Left*: Québec province; *Top right*: Focus on the largest cities of the province from Québec to Montréal; *Bottom right*: Focus on the Montréal and Laval regions

Figure 1 presents the location of healthcare establishments and facilities across the province of Québec (blue and black dots respectively) and the proportion of over 65-aged people in each local health networks (red gradation). Inserts on the right focus on the Montréal and Québec regions (top right) and the Montréal/Laval region (bottom right), for a clearer visualization of these zones. Table 1 presents the total number of individuals and people aged over 65, along with the healthcare offer and the main characteristics of each health region.

**Table 1:** Health facilities and population characteristics by health region (RSS)

Health region (RSS)	Number of local health networks	Number of facilities	Average number of facilities per local health network (min-max)	Average number of distinct services provided per facilities (min-max)	Total population	Over 65-aged population (%)	Number of dissemination area (DA)	Proportion of rural (DA)
01 - Bas-Saint-Laurent	8	78	9.8 (5–16)	1.6 (1–10)	202,062	60,709 (30%)	398	62.1%
02 - Saguenay-Lac-Saint-Jean	6	63	10.5 (7–13)	1.5 (1–10)	285,742	76,717 (26.8%)	526	40.2%
03 - Capitale-Nationale	4	130	32.5 (14–60)	1.6 (1–9)	792,370	187,337 (23.6%)	1,198	18.2%
04 - Mauricie et Centre-du-Québec	8	109	13.6 (4–27)	2.1 (1–10)	552,967	147,211 (26.6%)	917	40.4%
05 - Estrie	9	103	11.4 (3–29)	1.7 (1–6)	528,902	133,369 (25.2%)	798	52.2%
06 - Montréal	12	300	25 (7–39)	1.4 (1–6)	2,121,389	367,856 (17.3%)	3,185	0.3%
07 - Outaouais	5	69	13.8 (5–37)	1.7 (1–5)	418,749	81,433 (19.4%)	579	45.5%
08 - Abitibi-Témiscamingue	5	61	12.2 (10–13)	2 (1–7)	149,372	34,274 (22.9%)	298	54%
09 - Côte-Nord	7	54	7.7 (1–19)	2.1 (1–9)	89,682	20,943 (23.4%)	197	47%
11 - Gaspésie-Îles-de-la-Madeleine	5	47	9.4 (7–11)	2.4 (1–7)	92,809	29,782 (32.1%)	189	85.5%
12 - Chaudière-Appalaches	5	88	17.6 (4–46)	1.5 (1–7)	453,164	113,182 (25%)	723	46.2%
13 - Laval	1	44	44 (44–44)	1.2 (1–4)	456,705	90,400 (19.8%)	637	0%
14 - Lanaudière	2	67	33.5 (32–35)	1.4 (1–6)	557,613	120,201 (21.6%)	731	38.8%
15 - Laurentides	7	98	14 (5–28)	1.8 (1–11)	676,917	148,342 (21.9%)	872	40.6%
16 - Montérégie	9	193	21.4 (7–38)	1.4 (1–6)	1,507,246	318,417 (21.1%)	2,100	22.3%
**Québec province**	**93**	**1**,**504**	**16.57 (1–60)**	**1.65 (1–11)**	**8**,**885**,**689**	**1**,**930**,**173**	**13**,**348**	

There were 8,885,689 individuals in Québec in 2024, including 1,930,173 individuals aged 65 and over (21.7%) (Table 1). The proportion of older individuals in Québec varied between 17.3% and 32.1% at the regional scale and between 4.7% and 35.3% at the local scale. Interestingly, while only 17% of the Montréal region population was over 65 years old, 19% of the older population of the Québec province was in Montréal (Fig. 1; Table 1). In fact, the regions with the highest proportion of aged people within their own population were the Gaspésie (32.1%), Bas-Saint-Laurent (30%) and Saguenay (26.8%) regions, but the regions with the highest number of aged people in the province were Montréal (19.1%) and the Montérégie (16.5%). These two regions also had the highest overall population proportions of all ages (respectively 23.9% and 17%).

In 2024, the MSSS reported a total of 1,558 facilities under 131 health establishments located in 18 health regions and 93 local health networks in Québec province (Fig. 1; Table 1). A total of 1,504 facilities remained among the 93 local health networks after the exclusion of the regions of Nunavik, Terres-Cries-de-la-Baie-James and Nord-du-Québec due to missing data. At the region scale, the number of distinct facilities within the 15 remaining regions ranged from 44 (Laval region) to 300 (Montréal region), with a median of 74 facilities per health regions. Therefore, 19% of all facilities in Québec were in the Montréal region, followed by the Montérégie (12.4%). At the local scale, the number of facilities varied from 1 (“Port-Cartier”) to 60 (“Québec sud”) with a median of 13 facilities per local health networks.

### Services offered and urbanization level

**Fig. 2:**
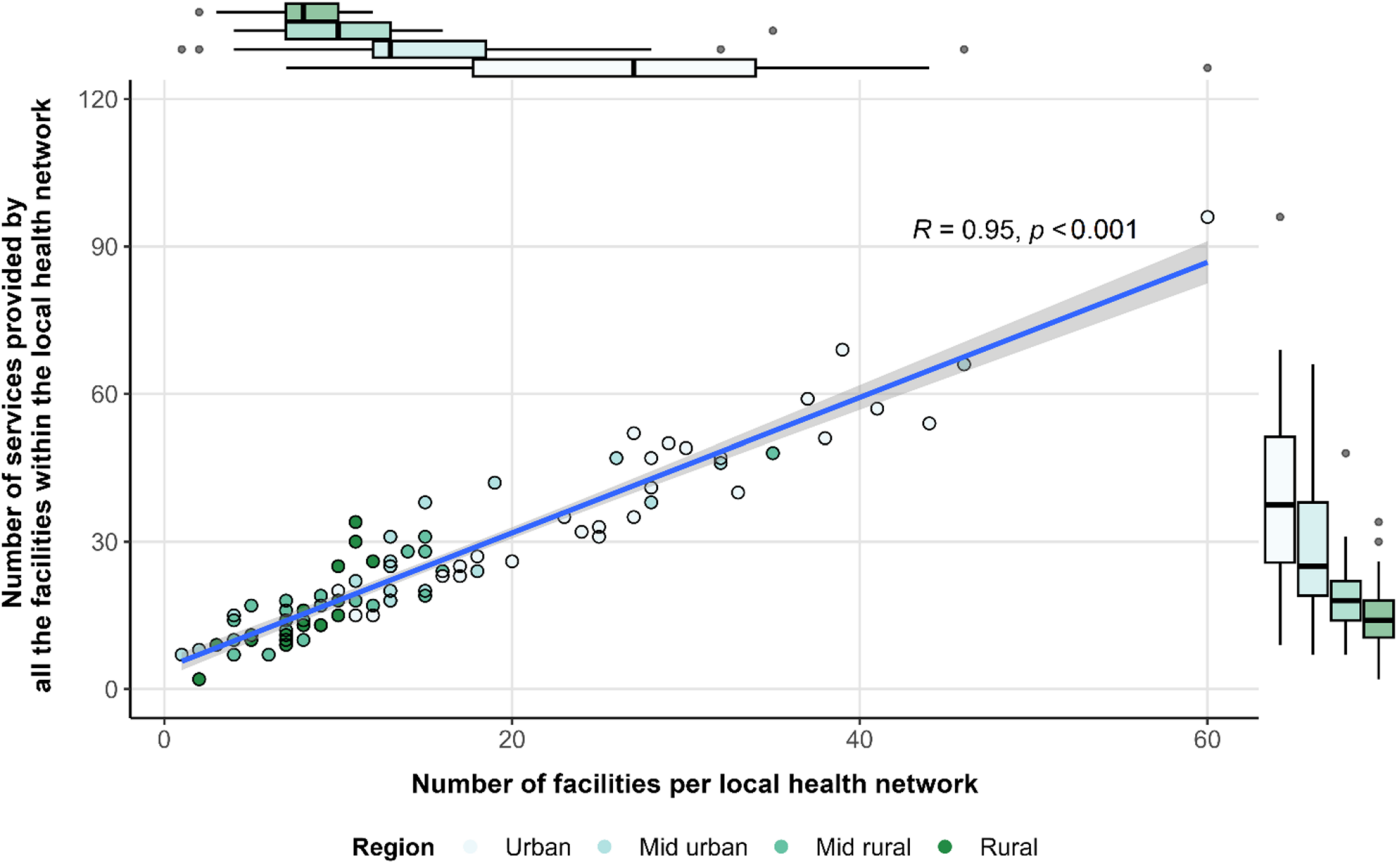
Number of installations and provided services per local health network and urbanization level. *Centre*: Number of services provided according to number of facilities per local health network. *The blue line* represents the smoothed conditional mean using the ‘y ~ x’ formula with its confidence interval. *Top and right*: related boxplot distribution according to the area’s level of urbanization. Boxes represent the interquartile range (IQR = Q3 -Q1), with the median shown as the central line. Whiskers extend to 1.5 x IQR, and dots indicate outliers as values that fall outside the lower and upper bound as Q1- 1.5 x IQR and Q3 + 1.5 x IQR

Understanding the variation in the number of services per facility helps capture differences in organization and specialization of healthcare resources across regions, which can influence the overall availability of services in urban and rural areas. Across Québec, facilities offered an average of 1.65 services, varying from 1 to 11 services (Table 1). Overall, 70% of facilities offered only 1 service. The characteristics of all the facilities in each local health networks were aggregated to present the results at this scale (Fig. 2). Therefore, in 50% of the 93 local health networks, there were at least 13 facilities, with an average of 16.2 facilities and 26.5 services per local health networks (median = 22). The local health network with the highest number of facilities was the “RLS Québec-sud” (*N* = 60), also offering the highest number of services (*N* = 96). The local health network with the lowest number of facilities was the “RLS de Port-Cartier” (*N* = 1), offering 7 services.

The total number of services offered by a local health network was directly correlated to the total number of facilities within that network (R^2^ = 0.95, *p* < 0.001) (Fig. 2, centre). However, there were slight differences in both the number of facilities (*p* < 0.001) and the offered services (*p* < 0.001) when considering the urbanization level of each local health networks (Fig. 2, boxplot on top and right side). Urban areas had significantly more services compared to rural areas, with an average of 35.2 services and 17.7 services respectively, when considering all services from all facilities within each local health networks. Interestingly, facilities within rural local health networks offered more services on average (1.8 services) compared to urban areas (1.6 services) (*p* < 0.001).

**Fig. 3:**
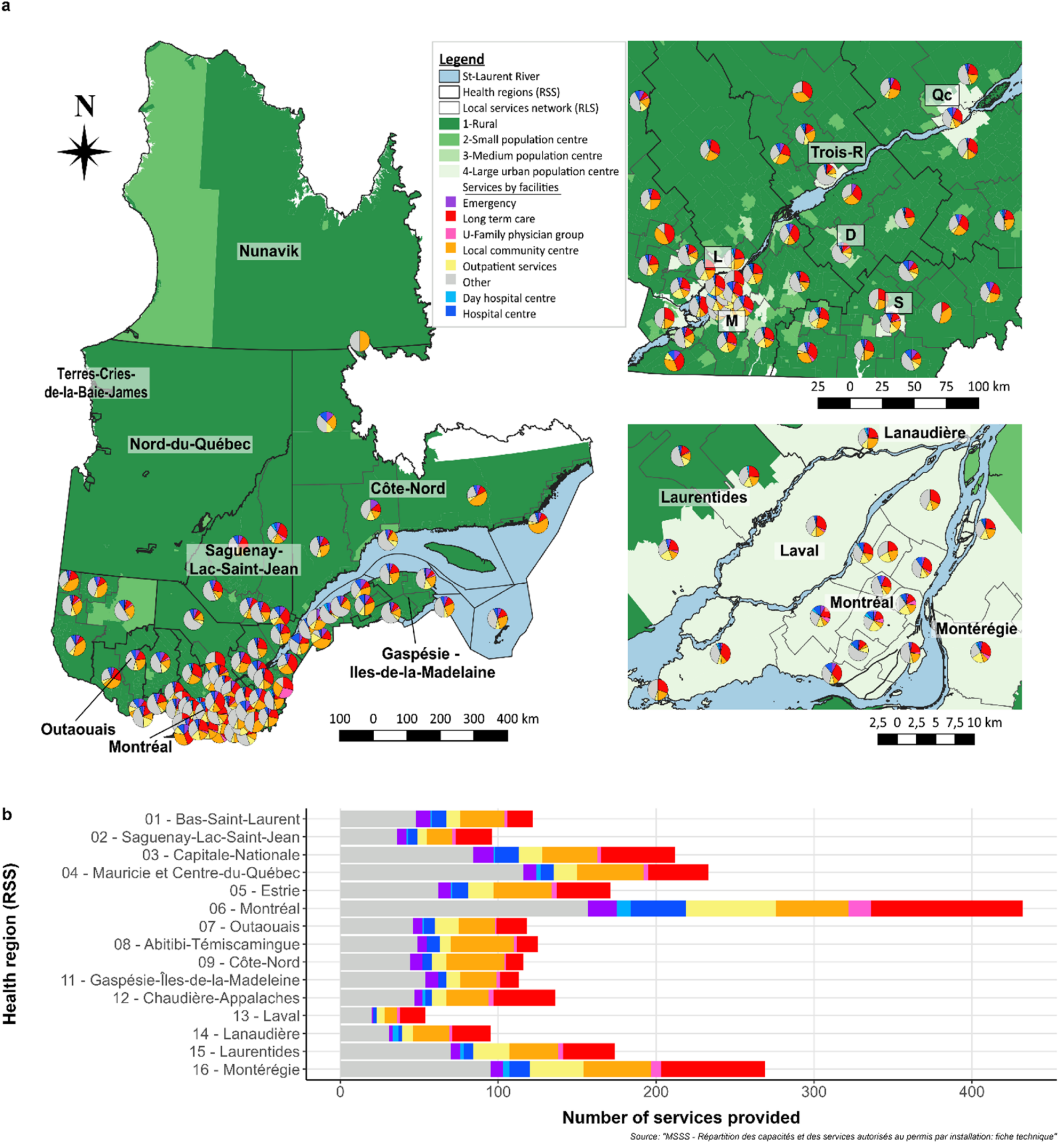
Services offer by health region and local health network. **a**) Rural and urban environment at the dissemination area scale and distribution of all services provided by the local health network. *Left*: Québec province; *Top right*: Focus on the largest cities of the province from Québec to Montréal; *Bottom right*: Focus on the Montréal and Laval regions. **b**) Number of different types of services offered by health region

The map in Fig. 3a presents the relative distribution of different types of services offered by each local health networks (pie chart) according to the level of urbanization (green gradation). Inserts on the right focus on the Montréal and Québec regions (top right) and the Montréal/Laval region (bottom right). The map is complemented by a cumulative bar plot (Fig. 3b), which provides further insight into the distribution of services across health regions. For example, Montréal, Capitale-Nationale, and Montérégie are urban regions (Fig. 3a), and we noted that the LTC accommodation was a service more frequently offered in these regions compared to rural areas, except for the Lanaudière region. Montréal was the region with the largest offer of all services, including the most LTC services (Fig. 3b). Additional maps of each health region and their level of urbanization are available in Online Resource 1.

### Long-term care accommodation and population location

**Fig. 4:**
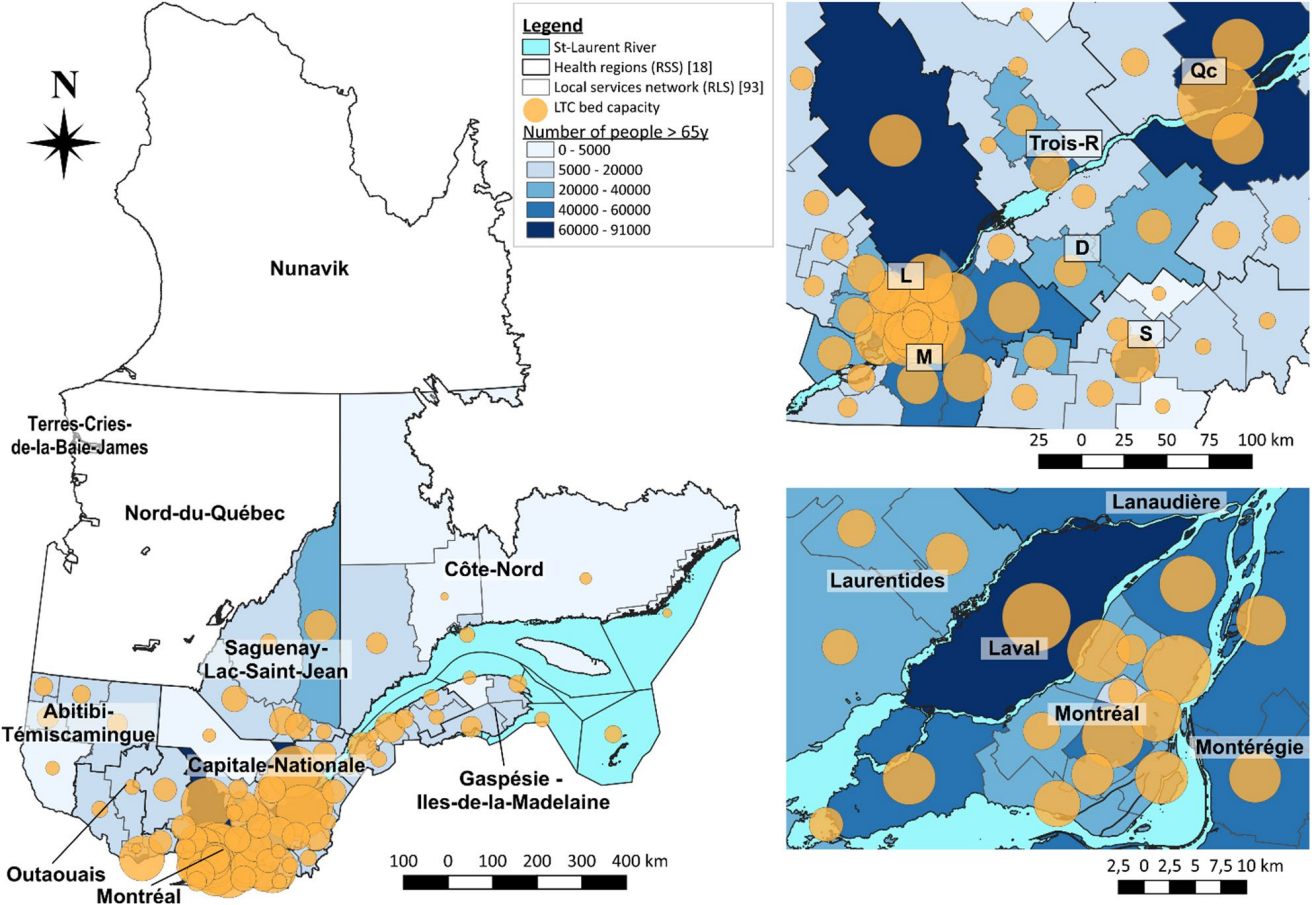
Number of people aged 65 and over and LTC beds at local health network scale. The actual number of the over 65-aged people at the local health network scale is represented with the number of beds available in LTC facilities for each health network (capacity). *Left*: Québec province; *Top right*: Focus on the largest cities of the province from Québec to Montréal; *Bottom right*: Focus on the Montréal and Laval regions

Figure 4 presents the number of over 65-aged people (blue gradient) and the LTC bed capacity of each local health regions across the province. Inserts on the right focus on the Montréal and Québec regions (top right) and the Montréal/Laval region (bottom right). A capacity of 44,989 LTC beds were found across the province, with a total of 23.3 beds per 1,000 people over 65 years old

**Fig. 5:**
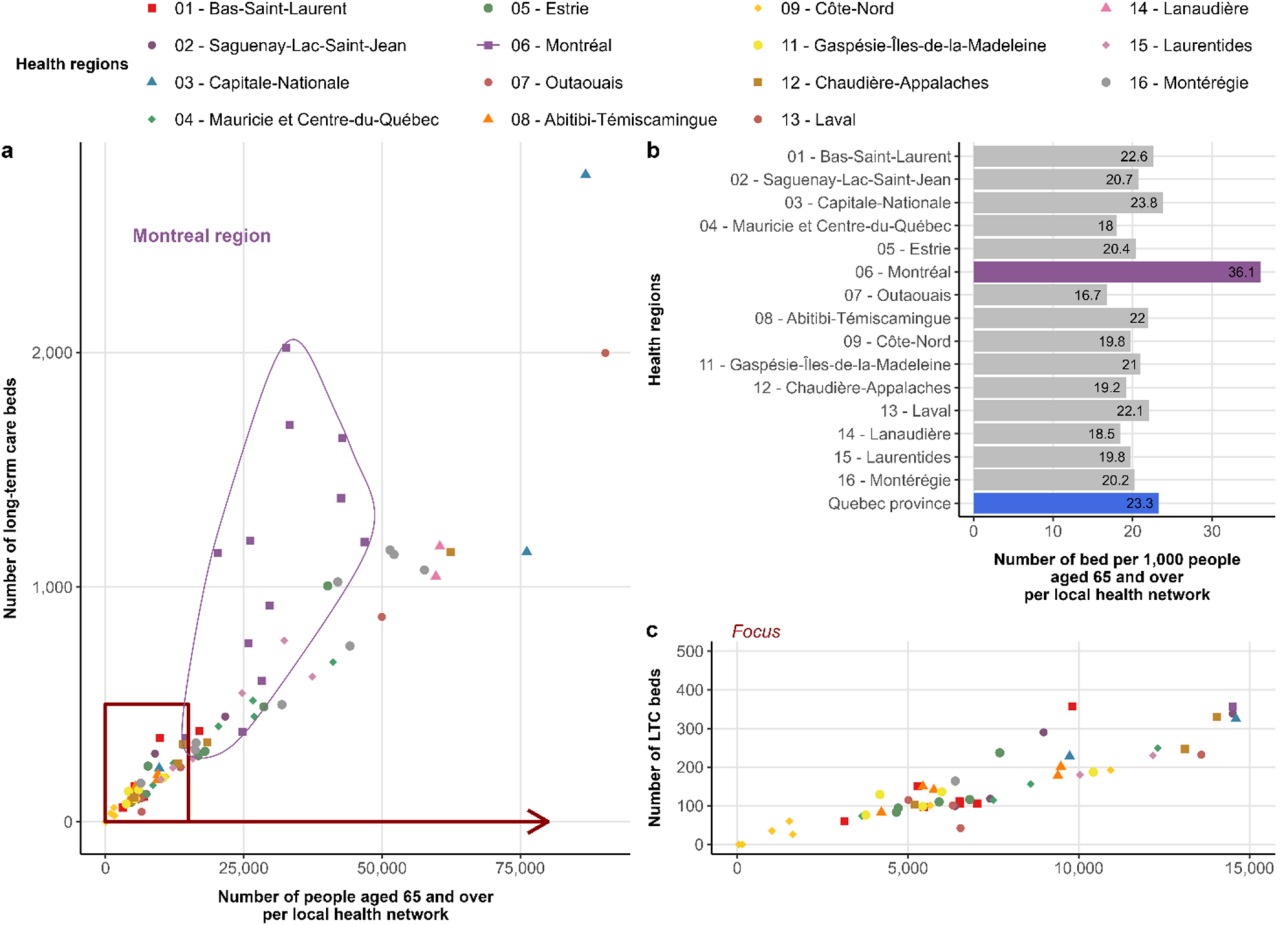
Long-term care beds and population at health region and local health network scales. **a**) Relation between number of long-term care beds and population density per local health network; **b**) Number of beds per 1,000 over 65-aged people; c) Focus of the relation between number of long-term care beds and population density per local health network from a)

At the regional scale, the number of beds varied between 16.7 and 36.1 beds per 1,000 people aged 65 and over, for Outaouais and Montréal regions respectively (Fig. 5b). At the local scale the number of beds per 1,000 people aged 65 and over varied between 6.4 and 61.8, for the RLS des Collines-de-l’Outaouais and RLS de Hochelaga - Mercier-Ouest - Rosemont respectively. A significant correlation was found between the number of beds and the number of over 65-aged people in the province (R² = 0.88, *p* < 0.001) (Fig. 5a and c). We identified the highest capacity for LTC beds in the Montréal region (36.1 beds per 1,000 over 65-aged people), where the proportion within the region was lower than in other regions but the absolute number of people over 65 was higher (Fig. 5b).

## Discussion

The objective of this study was to describe the geographical variation in the distribution of the aging population and the healthcare availability in Québec, Canada, using open access data. Across the province, the number of people aged 65 and over varied from 20,943 in Côte-Nord to 367,856 in Montréal. We noted that regions with the highest population overall also had the highest number of individuals aged 65 and over, namely Montréal and Montérégie. However, when considering the older individuals’ proportion in each region, the densely populated regions had the youngest average population. The proportion of older adults ranged from 17.3% in Montréal to 32.1% in Gaspésie and was 21.7% for the whole province. This interregional variation of the population aged 65 and over proportion was even more pronounced at the local scale, varying between 4.7% and 35.3%, highlighting an unequal distribution of the population across the local health networks, which may translate into unequal healthcare needs across territories, as suggested in previous studies [4, 9].

Furthermore, we observed a substantial variability of the number of facilities at both the regional scale (from 44 to 300) and the local scale (from 1 to 60), with a high degree of specialization as 70% of facilities offered only one service. In the Québec province, we found that the healthcare availability was associated with the number of older people. The regions with the highest number of facilities and services, including LTC services, were also the ones with the highest number of older individuals. For example, the Montréal region presented 19% of all facilities in Québec. Similarly, we observed a variability in the number of LTC beds at both the regional and local scale and found a significant correlation between the number of LTC beds and the number of over 65-aged people across the province (R² = 0.88, *p* < 0.001). It is crucial to consider LTC capacity as it can be considered as the most offered and necessary service to older adults, along with homecare. Indeed, LTC accommodation play a key role in supporting individuals with significant loss of autonomy, particularly when living at home is no longer viable. In 2016, 4.39% of people aged 65 and over in Québec were residents in LTC facilities [34]. Overall, the province of Québec had a capacity of 23.3 beds per 1,000 over 65-aged people at the time of the study. These findings align with those presented by Canadian Institute for Health Information (CIHI), which reported 24 LTC beds per 1,000 older adults in Québec in 2021 [35]. These results suggest a proportional allocation of healthcare resources across regions. However, this apparent alignment does not necessarily guarantee adequacy in meeting population needs, as the available resources may not be sufficient to meet the complex and growing needs of the aging population. For instance, CIHI’s data indicate that Québec’s ratio remains below the national average of 29 LTC beds per 1,000 older adults [35]. This comparison should nonetheless be interpreted with caution, given interprovincial differences in care delivery models. Moreover, no evidence-based optimal ratio of LTC beds exists, as such a ratio depends on multiple factors, including the availability of alternative services.

To better understand the differences between local health networks, we considered the level of urbanization. There were slight differences in both the number of facilities (*p* < 0.001) and the services offered (*p* < 0.001) when considering the urbanization level of each local health networks. As hypothesized, urban areas had substantially more services offered compared to rural areas, (average of 35.2 and 17.7 services respectively) when considering all services from all facilities within each local health networks. The lower healthcare availability in rural areas has also been documented in the literature in Manitoba and the USA [9, 23]. This disparity is significant considering that 25% of the population lived in rural areas in Québec in 2021 [22]. Moreover, LTC accommodation is a service most offered in urban rather than rural areas, suggesting that older population living in rural areas would have fewer LTC services in their environment than urban residents. As previously noted, rural regions are also those where population aging is most advanced, meaning that these areas may face specific vulnerabilities. This could potentially lead to substantial care needs that exceed local capacity. This disparity could raise concerns as studies already demonstrated that hospitalization outside of the region of residence can have serious consequences for the older adults [18, 19]. This descriptive study, complemented with more detailed information on services such as home care, could support the identification of regions in Québec that lack sufficient healthcare resources, and help guide preliminary reflections on how to better organize resources according to local demands.

However, the number of services does not necessarily imply adequate access to care, as demonstrated by Montréal. Montréal is a prominent example of an urban area, accounting for 19% of the Québec older population and with the highest number of facilities, services and LTC beds capacity (36.1 per 1,000 habitants). These findings align with the CSBE study, which reported that in 2019–2020, Centre-Sud-de-l’Ile-de-Montréal was the local health network with the highest number of public LTC centres [36]. However, a study from 2010 showed that aged people have a low level of access to healthcare facilities due to their lower level of mobility [20]. More globally, another study from 2018 demonstrated that access and equity issues existed, especially for the older adults in Québec [37]. These results reinforce the need for future research to consider other factors in addition to availability to better understand healthcare access for this population.

### Strengths and limitations

The main strength of this study lies in the use of reliable, open-access data sources, which ensures representativeness across regions of Québec. Nonetheless, a few limitations should be acknowledged. First, by relying solely on open access data, we represented the demand for healthcare by the number and location of over 65-aged people. This approximation has limitations as not all individuals in this age group necessarily have healthcare needs, and not all needs result in expressed demand. The analysis would have been enhanced by incorporating additional factors such as clinical status, quality of life, or lifestyle to better capture the healthcare demand. While such data were not available for this study, future spatial analyses could benefit from the integration of these dimensions, particularly by leveraging the potential of Geographic Information Systems (GIS). GIS tools allow for the combination of spatial data with individual-level or contextual variables, to better identify geographic barriers and potential inequities in access to care. Furthermore, research has shown that increasing material and social deprivation is associated to a higher prevalence of multimorbidity [4]. It would have been beneficial to incorporate the deprivation index, which was developed to document disparities in health according to the population level of resources [38]. However, due to the lack of precise information on individuals’ residential locations, it was not possible to include this factor.

Secondly, available healthcare was represented by the facilities location, and the services and number of beds provided. While this approach provides a general overview of the distribution of healthcare availability, the lack of real-time bed availability, human resources information and detailed utilization data in publicly available sources limit the ability to capture more nuanced dynamics, such as potential compensations between service types (for example, LTC and home care). In addition, healthcare services are usually planned to serve the entire population, not only older adults. As such, the number of services could be more closely related to the general population needs. However, to partially address this limitation, we deliberately focused on services primarily targeted to people aged 65 and over. Finally, it is important to note that three northern regions were excluded from the analysis due to missing data. These regions are known to face unique challenges in healthcare availability and access [39]. Including them in future analyses would help capture a more comprehensive portrait of regional disparities across Québec.

## Conclusion

The objective of this study was to describe the existing variation in older population location and healthcare availability in Québec, Canada, using open access data. We found that the healthcare availability in Québec is associated with the number of people aged 65 and over and that there tend to be fewer services overall and per older person in rural as compared to urban areas. This finding suggests that older adults living in sparsely populated areas will have less access to a variety of services, including LTC. Consequently, we identified a substantial variability in population’s location and in the number of facilities and services provided in Québec, especially at the local scale. These findings contribute to filling a gap in knowledge on healthcare services availability in Québec and may orient future work exploring how healthcare resources relate to population needs.

## Supplementary Information

Below is the link to the electronic supplementary material.

Supplementary Material 1

## Data Availability

The datasets analysed during the current study are publicly available. Generated datased are available from the corresponding author.

## Abbreviations

LTC: Long-Term Care
MSSS: Ministère de la Santé et des Services sociaux [Ministry of Health and Social Services]
RLS: Réseaux Locaux de Services [Local health networks]
RSS: Régions Sociosanitaires [Health regions]
