# Supplementary material for: Spatial analysis of healthcare services availability and demand for people aged 65 and over in Québec
Source: Res Health Serv Reg. 2026 Jan 21;5:2. doi: 10.1007/s43999-026-00085-5 (PMC12824029; doi:10.1007/s43999-026-00085-5)
Supplement: Supplementary file 1 — Supplementary Material 1 [file 43999_2026_85_MOESM1_ESM.pdf]

Spatial analysis of healthcare services availability and demand for people aged 65 and over in Québec

Research in Health Services & Regions

Juliette Duc[1,2,3], Nevena Veljanovic[1], Sébastien Barbat-Artigas[4], David L. Buckeridge[5], Delphine Bosson-Rieutort[1,2,3]

[1] Department of Health Management, Evaluation and Policy, School of public health, Université de Montréal, Montreal, Qc, Canada

[2] Centre de recherche en santé publique (CReSP), Université de Montréal et Centre intégré universitaire de santé et de services sociaux du Centre-Sud-de-l'Île-de-Montréal, Montréal, Québec, Canada

[3] Centre Interuniversitaire de Recherche en Analyse Des Organisations (CIRANO), Montréal, Québec, Canada

[4] Unité d'évaluation des technologies et modes d'intervention en santé et services sociaux (UETMIS-SS), Direction des affaires universitaires, de l'enseignement et de la recherche (DAUER), Centre intégré universitaire de santé et de services sociaux de l'Ouest-de-l'île-de-Montréal (CIUSSS-ODIM)

[5] Department of Epidemiology, Biostatistics and Occupational Health, McGill University, Montreal, Canada

Corresponding authors:

- Juliette Duc

juliette.duc@umontreal.ca

- Delphine Bosson-Rieutort

delphine-bosson-rieutort@umontreal.ca

Services offer by each local health network within health region (RSS). Relative distribution of different types of services offered by each local health networks (pie chart) according to the level of urbanization (green gradation).

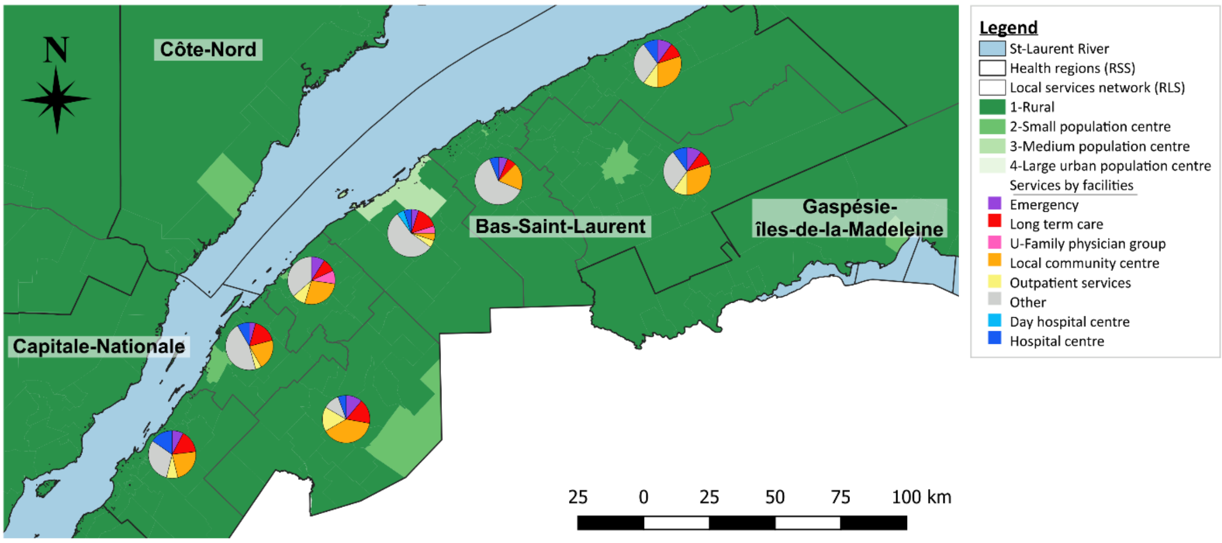

RSS 1: Bas-Saint-Laurent

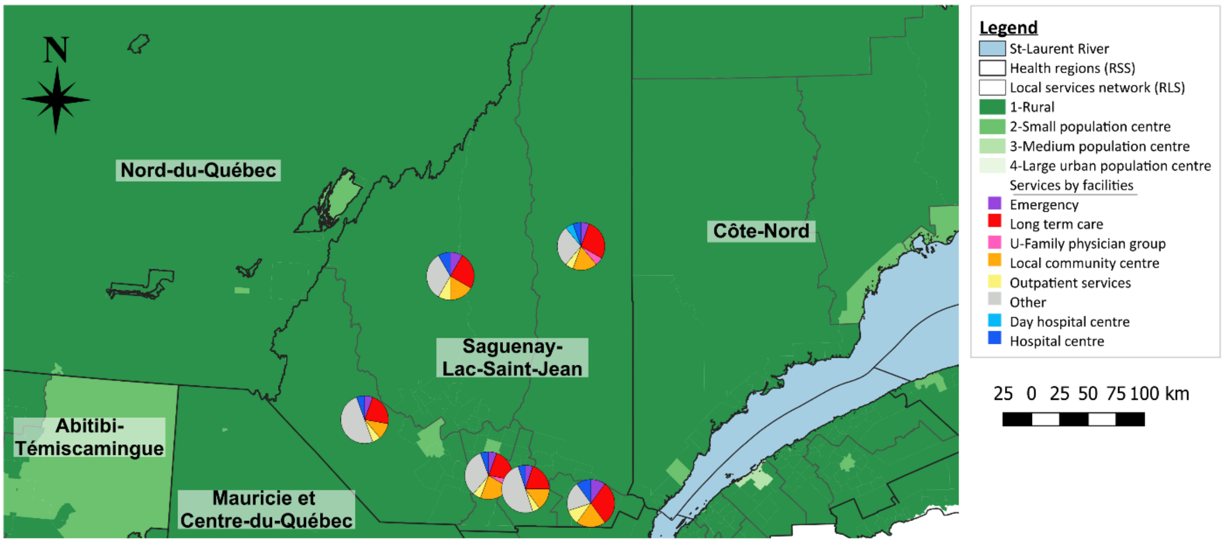

RSS 2: Saguenay – Lac-Saint-Jean

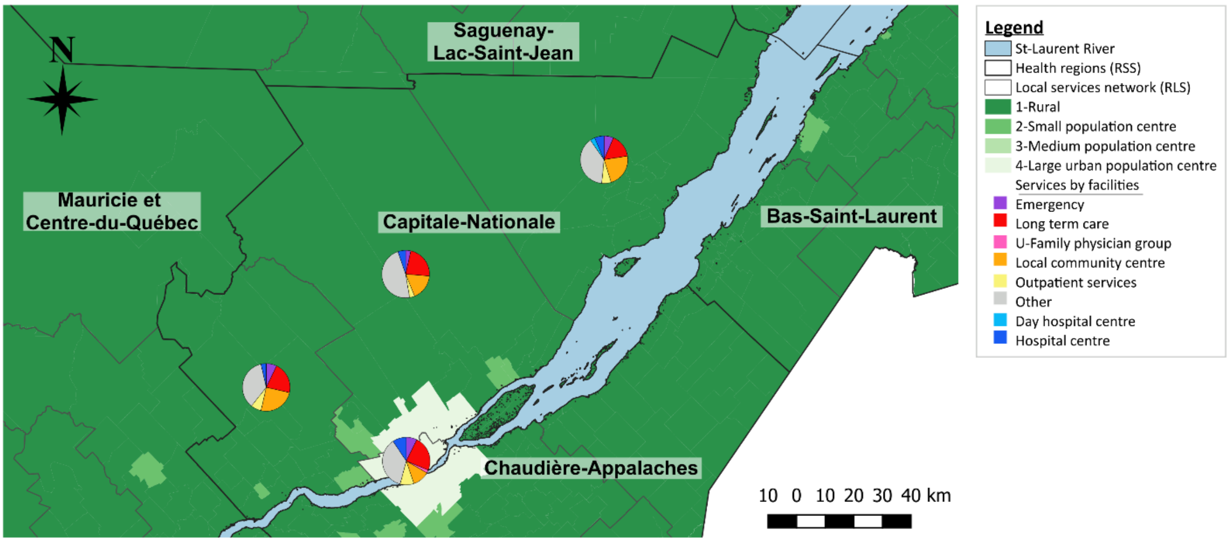

RSS 3: Capitale-Nationale

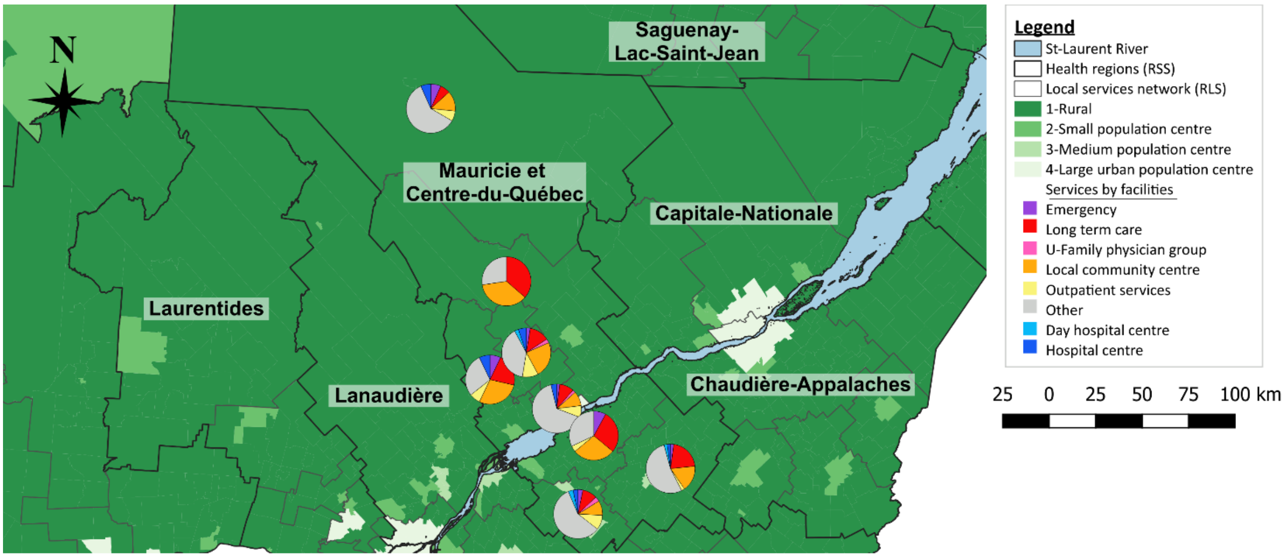

RSS 4: Mauricie and Centre-du-Québec

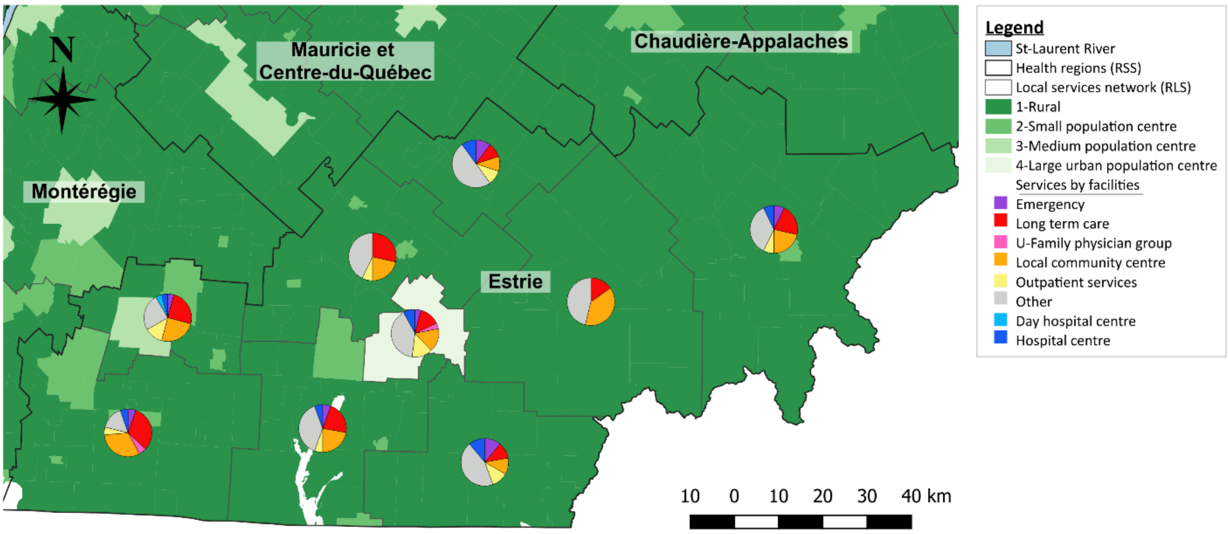

RSS 5: Estrie

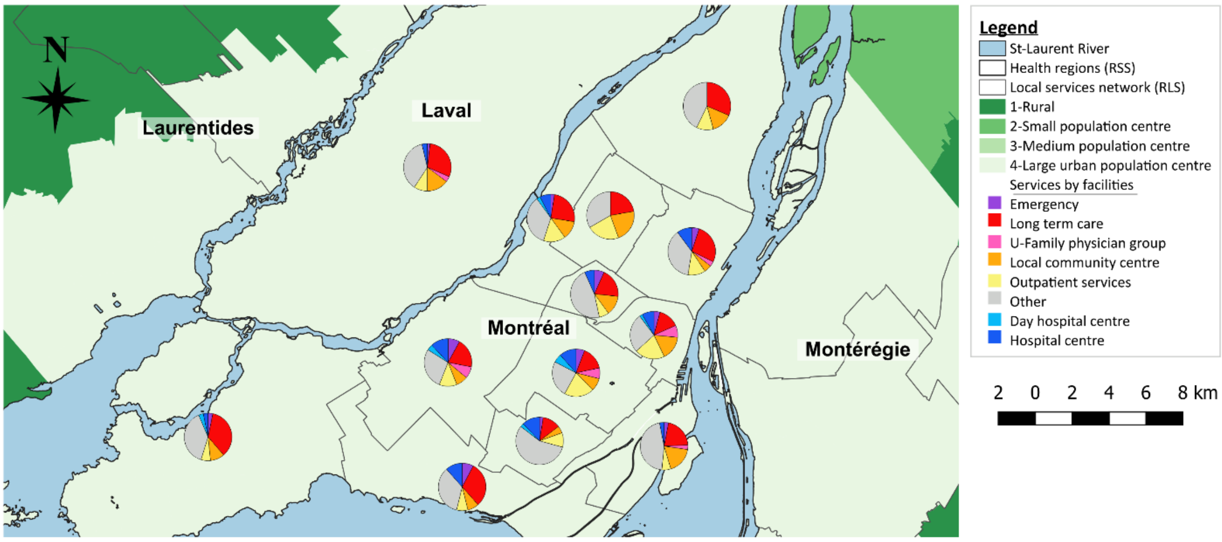

RSS 6: Montréal and RSS 13: Laval

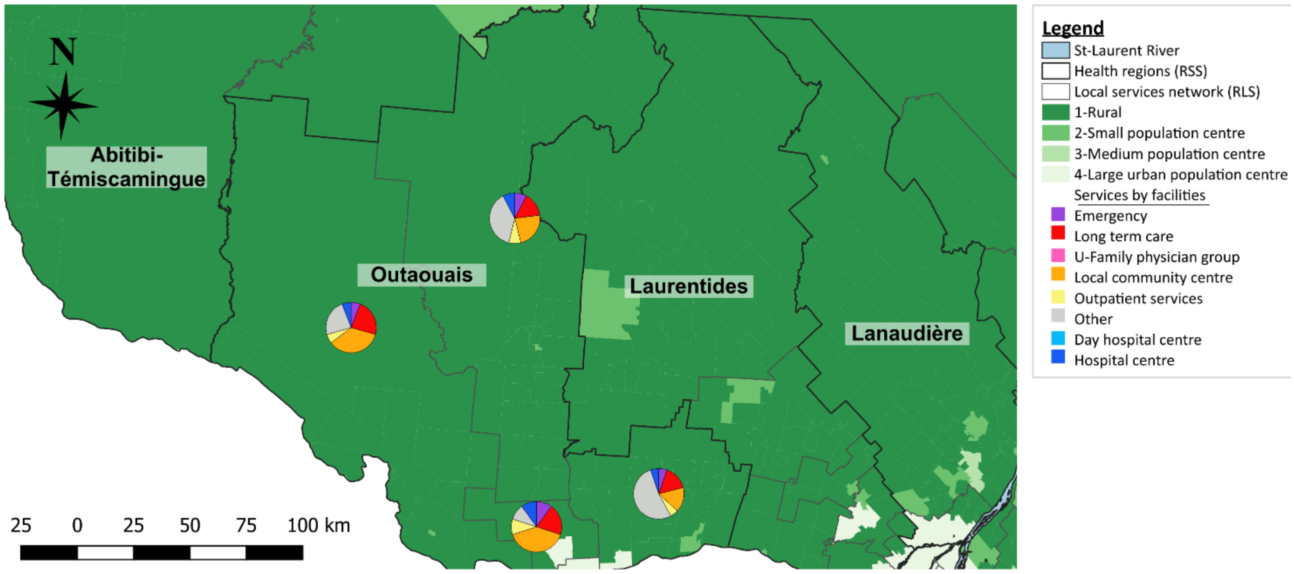

RSS 7: Outaouais

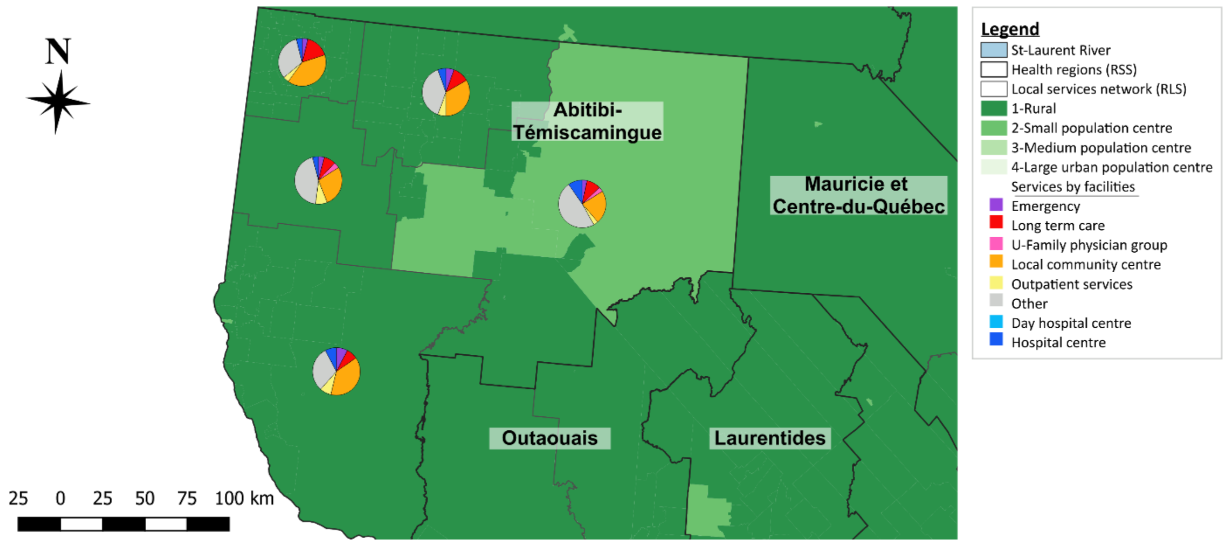

RSS 8: Abitibi-Témiscamingue

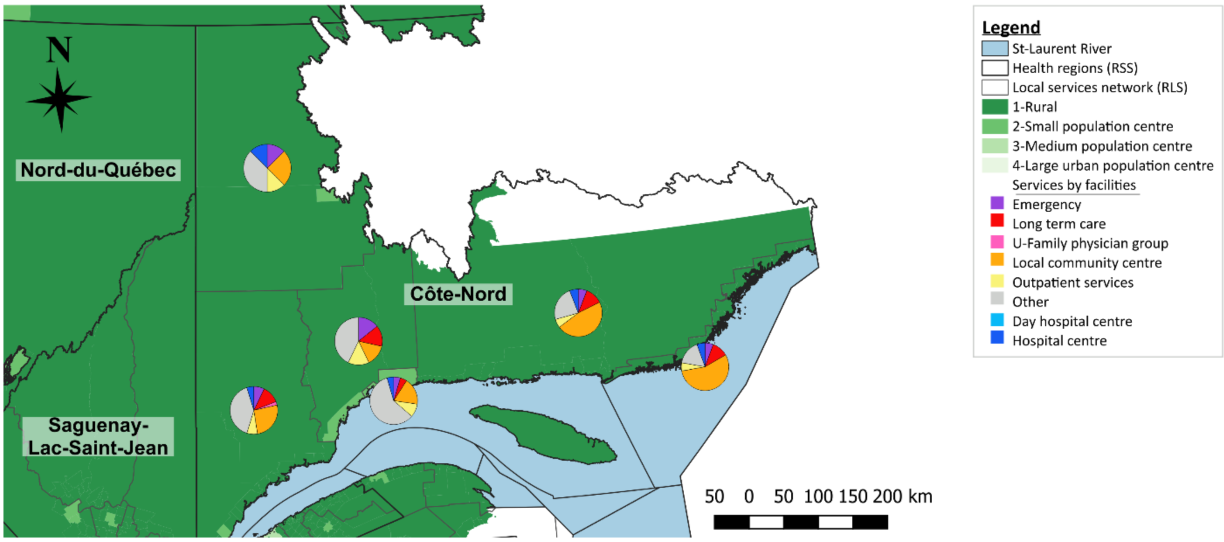

RSS 9: Côte-Nord

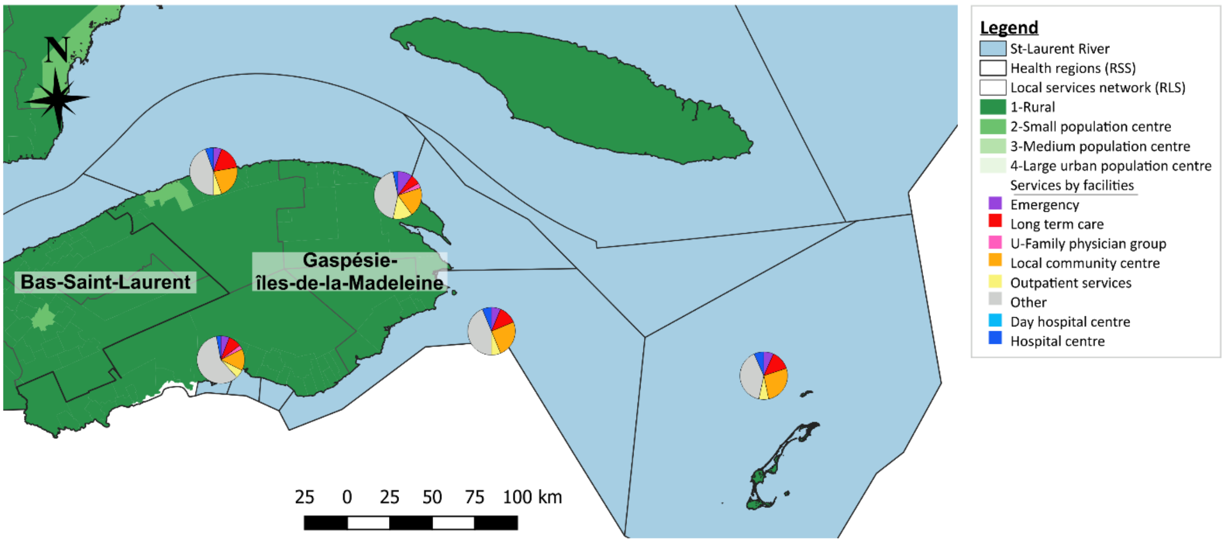

RSS 11: Gaspésie-Îles-de-la-Madeleine

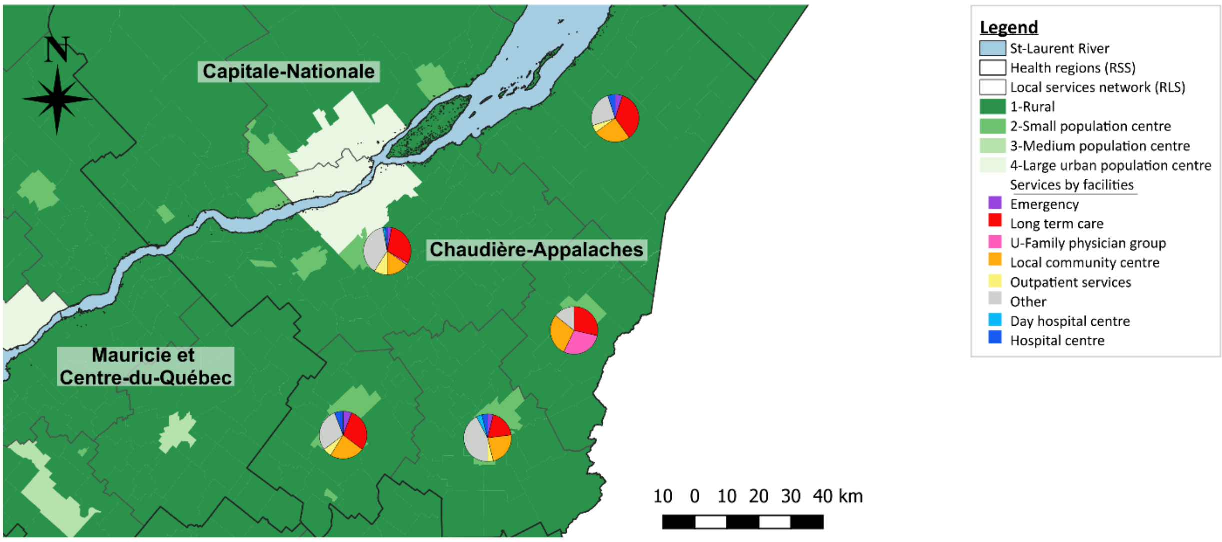

RSS 12: Chaudière-Appalaches

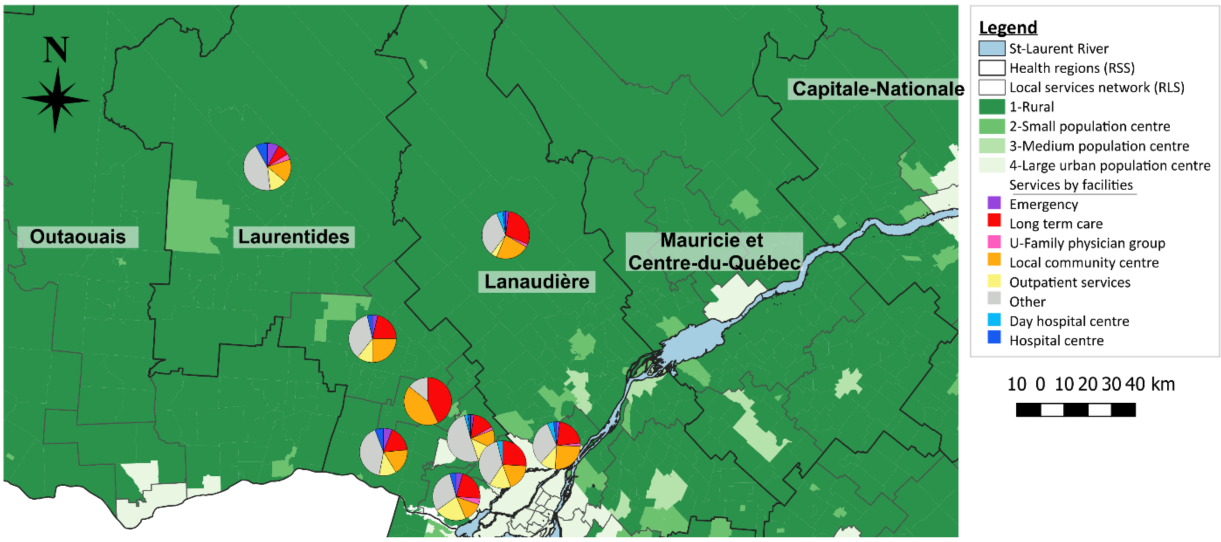

RSS 14: Lanaudière and RSS 15: Laurentides

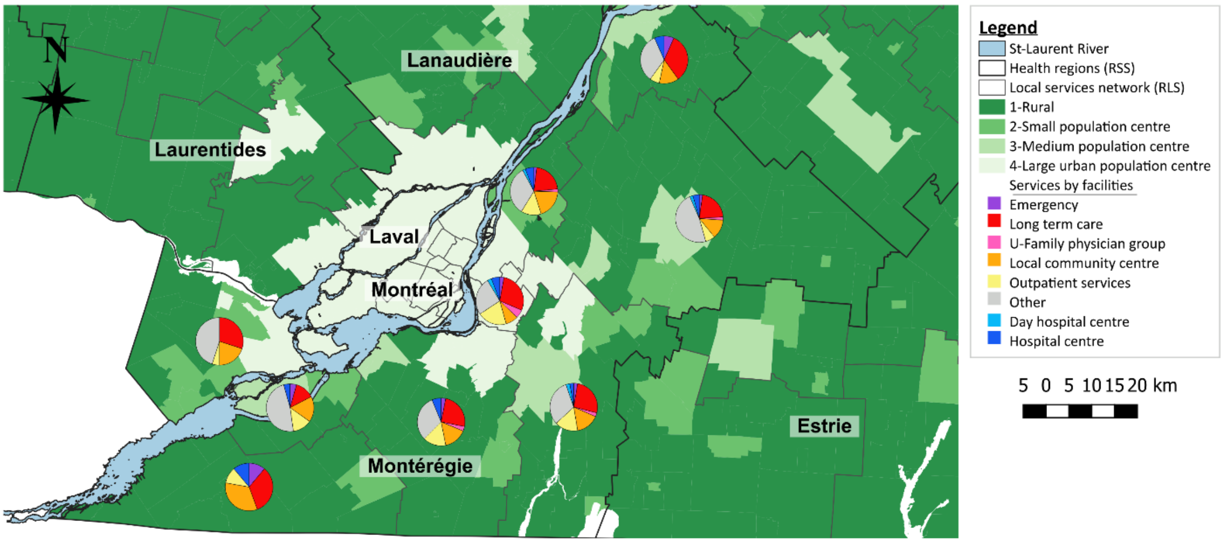

RSS 16: Montérégie
